# Author Correction: Role of Tim4 in the regulation of ABCA1^+^ adipose tissue macrophages and post-prandial cholesterol levels

**DOI:** 10.1038/s41467-022-29352-y

**Published:** 2022-03-25

**Authors:** M. S. Magalhaes, P. Smith, J. R. Portman, L. H. Jackson-Jones, C. C. Bain, P. Ramachandran, Z. Michailidou, R. H. Stimson, M. R. Dweck, L. Denby, N. C. Henderson, S. J. Jenkins, C. Bénézech

**Affiliations:** 1 Centre for Cardiovascular Science, University of Edinburgh, Edinburgh, UK; 2 Centre for Inflammation Research, University of Edinburgh, Edinburgh, UK; 3 Division of Biomedical and Life Sciences, Lancaster University, Lancaster, UK; 4 MRC Human Genetics Unit, Institute of Genetics and Molecular Medicine, University of Edinburgh, Edinburgh, UK

**Keywords:** Monocytes and macrophages, Fat metabolism, Dyslipidaemias

Correction to: *Nature Communications* 10.1038/s41467-021-24684-7, published online 21 July 2021.

In this article the author name Zoi Michailidou was incorrectly written as Zoi Michalidou. The original article has been corrected.

