# Supplementary material for: Role of Tim4 in the regulation of ABCA1+ adipose tissue macrophages and post-prandial cholesterol levels
Source: Nat Commun. 2021 Jul 21;12:4434. doi: 10.1038/s41467-021-24684-7 (PMC8295389; doi:10.1038/s41467-021-24684-7)
Supplement: Supplementary file 1 — Supplementary Information [file 41467_2021_24684_MOESM1_ESM.pdf]

Role of Tim4 in the regulation of ABCA1^{+} adipose tissue macrophages and post-prandial cholesterol levels. Magalhaes et al.

Supplementary Materials

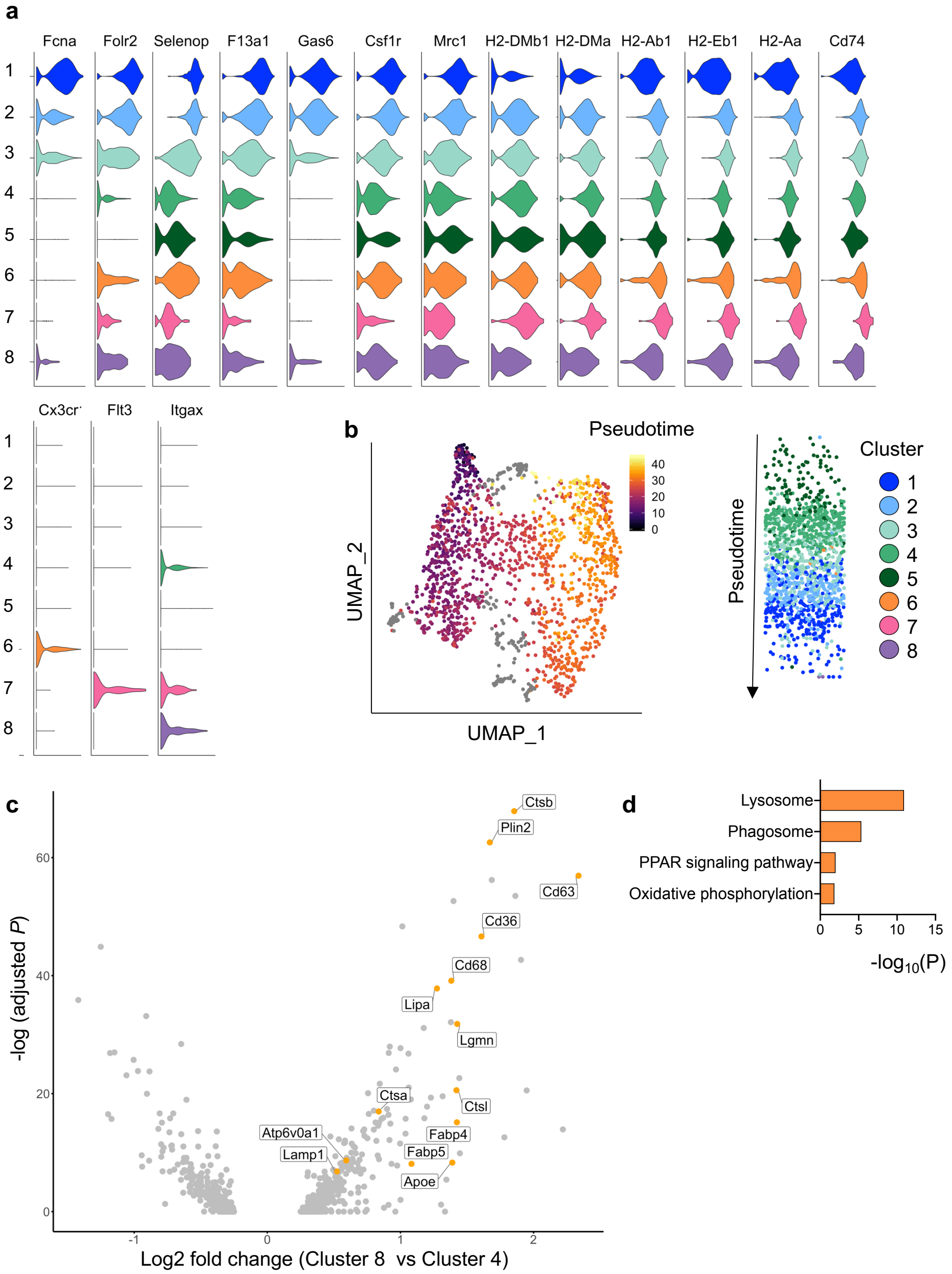

Fig. S1. Characterization of ATM clusters. a Violin plots of canonical ATM gene expression by cluster. **b** Slingshot analysis of ATM trajectory in mice fed overnight HFD. UMAP visualization of the Pseudotime values with Cluster 5 as starting point. **c** Volcano plot showing DEGs between cluster 8 (LAM ATMs) and cluster 4 (BM derived ATMs). Examples of DEGs distinguishing cluster 8 are shown in orange. **d** KEGG Pathway analysis on DEGs distinguishing cluster 8.

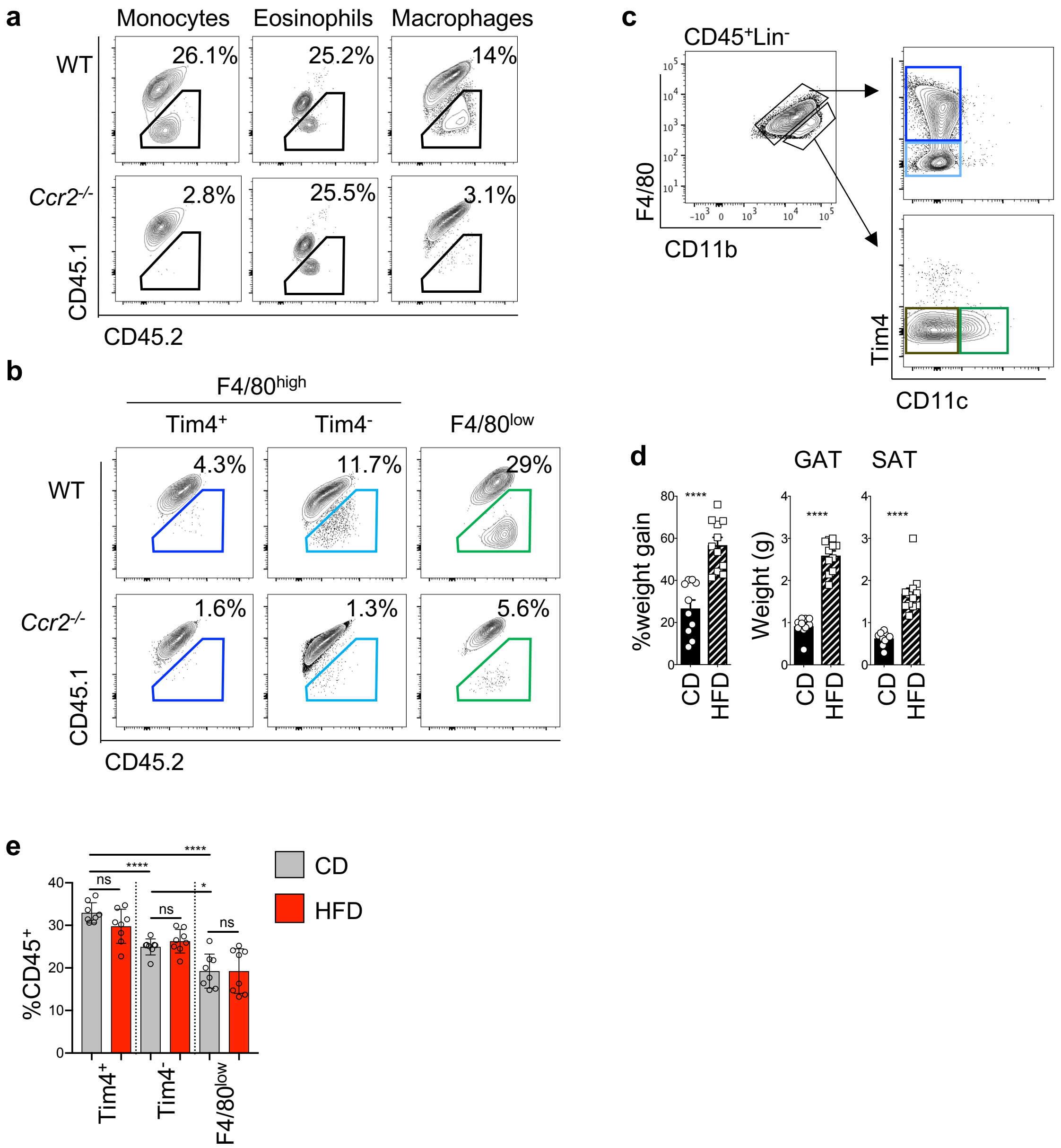

Fig. S2. Analysis of non-host chimerism in protected BM chimeras. a, b Flow-cytometric analysis of epididymal AT showing contour-plots and the gating of non-host cells in monocytes, eosinophils and macrophages (**a**) and in the indicated ATM subsets (**b**). **c** Flow-cytometric analysis of epididymal AT showing the gating of ATM subsets. **d** Weight gain of mice kept on CD or HFD and weight of their epididymal AT (EAT) and subcutaneous AT (SAT). Data pooled from 2 independent experiments with n=10 mice per group. **e** Quantification of the percentage of the indicated ATM subsets found in the $CD45^+$ cell fraction in mice kept on CD diet (grey) or fed HFD overnight (red). Data pooled from 2 independent experiments with n=8 mice per group. Error bars show SEM. Kruskal Wallis test with Dunn's multiple comparisons test or ANOVA with Sidak's multiple comparisons test were applied after assessing normality using D'Agostino and Pearson Normality test (**e**). Two-tailed Student's T-test was applied in **d**. Significant differences are indicated by **** P =<0.0001, * P=<0.05, ns non-significant.

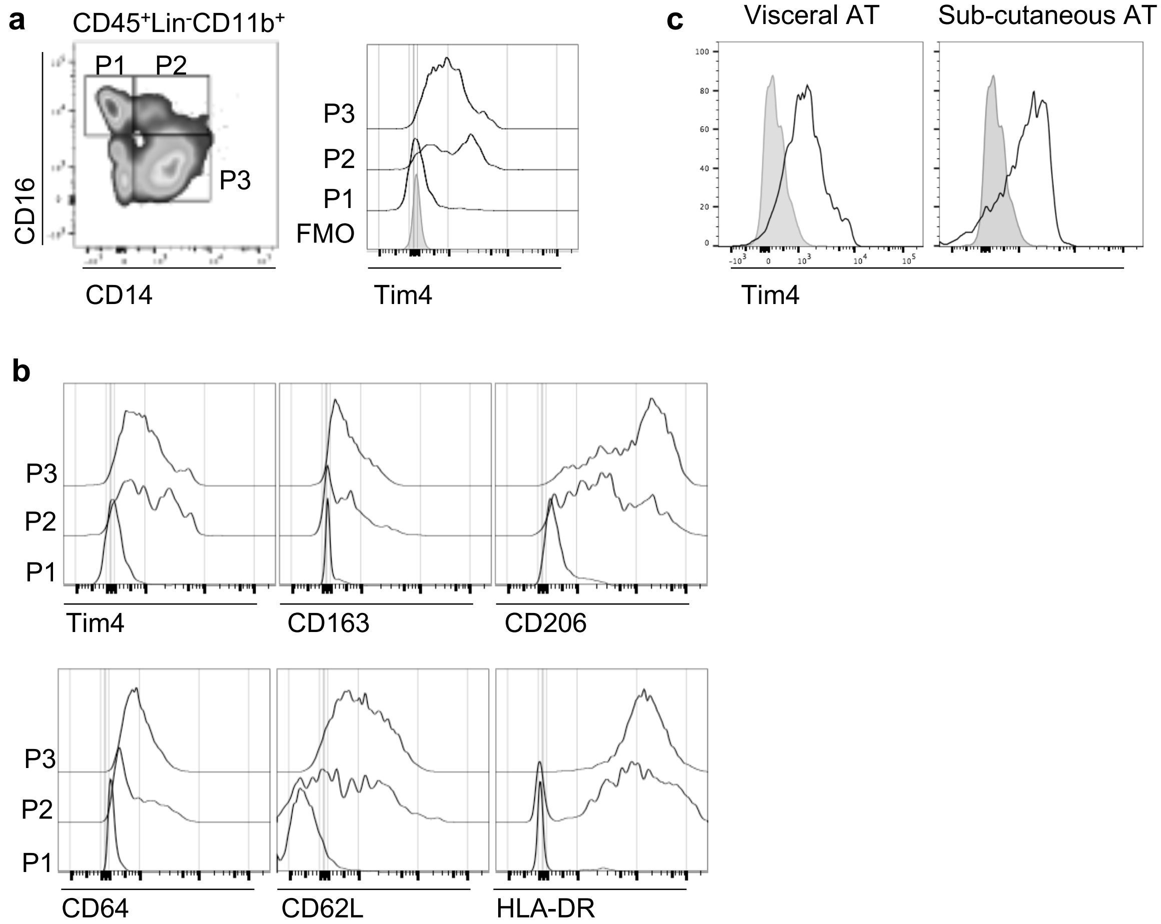

Fig. S3. Analysis of human ATMs. **a** Flow-cytometric analysis of ATMs in human omental AT with representative density plot of $CD45^{+}Lin^{-}CD11b^{+}$ cells showing $CD16^{+}CD14^{-}$ ATMs (P1), $CD16^{+}CD14^{+}$ ATMs (P2) and $CD16^{-}$ $CD14^{+}$ ATMs (P3) and histogram showing Tim4 expression in P1, P2 and P3. Fluorescence minus one (FMO) in grey. Lineage (Lin) includes CD19, CD3 and NCAM. **b** Histograms showing expression of common macrophage markers on the 3 subsets. **c** Histograms showing the expression of Tim4 on $CD45^{+}Lin^{-}CD11b^{+}$ ATMs in the omental and subcutaneous AT. Data representative of 3 patients.

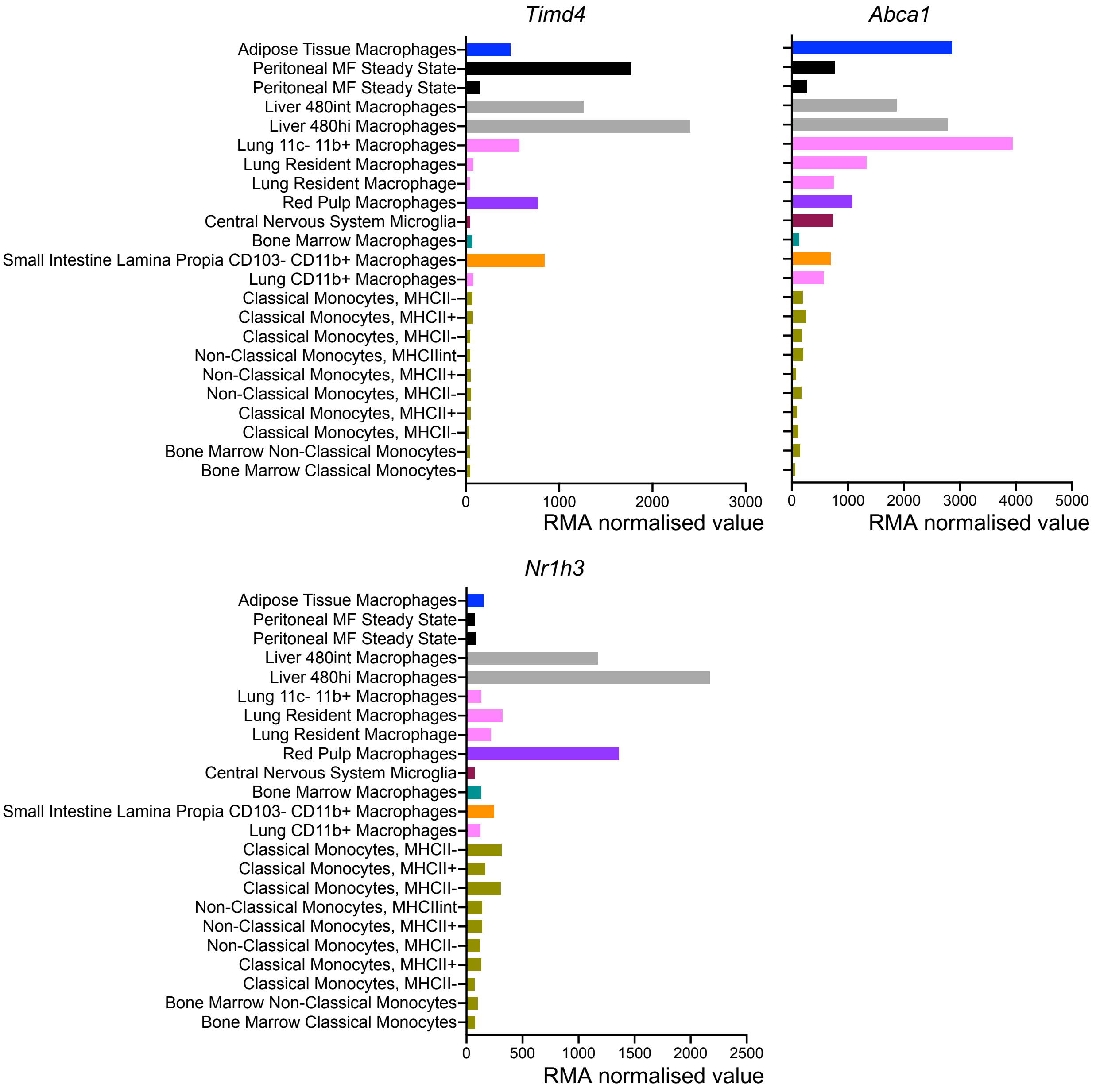

Fig. S4. Expression of *Timd4*, *Abca1* and *Nr1h3* in macrophages and myeloid cells from different tissues. Normalized expression of *Timd4* (left), *Abca1* (right) and *Nr1h3* (lower left) obtained from the ImmGen microarray dataset in ATM (blue) and peritoneal (black), liver (grey), lung, red pulp, microglia, bone marrow and lamina propria macrophages, as well as monocytes.

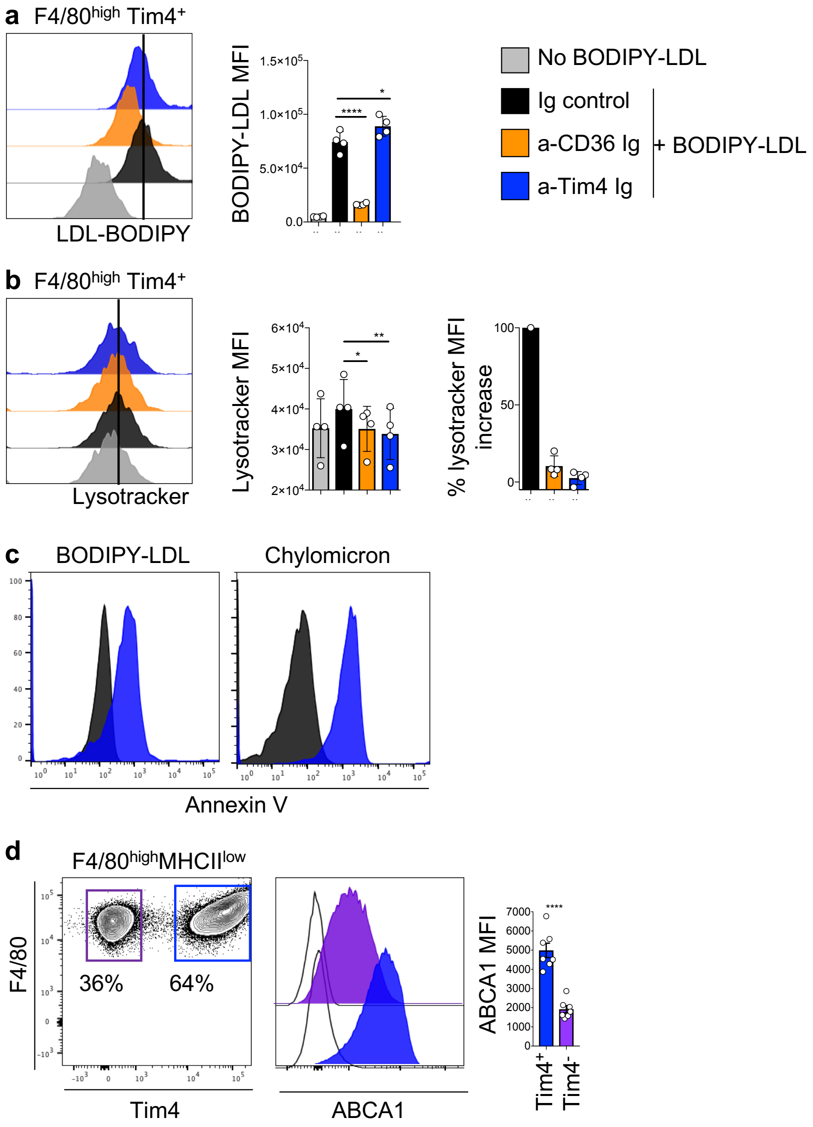

Fig. S5. CD36 regulates LDL uptake by F4/80^{high}Tim4^{+} ATMs. Adipose tissue cells were pre-incubated with blocking anti-CD36 IgG or blocking anti-Tim4 Ig or IgG control prior to incubation with BODIPY-LDL for 1 hour. **a, b** Flow-cytometric analysis showing histogram of BODIPY-LDL (**a**) and lysotracker (**b**) fluorescence intensity in F4/80^{high}Tim4^{+} ATMs, quantification of mean fluorescence intensity (MFI) of BODIPY-LDL and lysotracker and percentage increase in lysotracker MFI compared to no LDL-BODIPY. Cells with no LDL-BODIPY (grey), incubated 1 hour with LDL-BODIPY and Ig control (black), anti-CD36 (orange) or anti-Tim4 Ig (blue). To allow detection of Tim4 in cells incubated with anti-Tim4 Ig, Tim4 was detected using rat anti-Tim4 Ig plus secondary anti-Rat Ig-647. Data representative of 2 experiments with n=4 mice per group. **c** Flow-cytometric analysis showing histogram of Annexin-V of BODIPY-LDL and chylomicrons. **d** Flow-cytometric analysis showing gating of F4/80^{high}Tim4^{+} and F4/80^{high}Tim4^{-} peritoneal macrophages, histogram of ABCA1 and quantification of ABCA1 MFI in both macrophage populations. Data pooled from 2 independent experiments with n=8 mice per group. Error bars show SEM. Student's paired T-test (**a, b**), Two-tailed Student's T-test (**d**). Significant differences are indicated by * P=<0.05, ** P=<0.01, **** P =<0.0001.

Antigen Name	Conjugate	Clone	Manufacturer
**Anti-mouse antibodies**			
ABCA1	Alexa-647	MCA2681A647	Bio-Rad
CD11b	PE Dazzle	M170	Biolegend
	VioBlue	REA592	Miltenyi
CD11c	BV605	N418	Biolegend
	APC-Vio770	REA754	Miltenyi
CD115 (CSF1R)	APC		Biolegend
CD19	BV421	6D5	Biolegend
CD36	PE	REA1184	Miltenyi
CD206	FITC	MCA2235FA	Miltenyi
CD45.1	PE/Cy7	A20	Biolegend
CD45.2	BV450	104	eBiosciences
	BV650	104	Biolegend
F4/80	PE/Cy7	BM8	Biolegend
	FITC	REA126	Miltenyi
HSP-70 (HSP1A1)	FITC	REA349	Miltenyi
Ki-67	FITC	REA183	Miltenyi
LAMP1	PE	REA792	Miltenyi
LAMP2	PE	M3/84	Miltenyi
Ly6C	AF700	HK1.4	Biolegend
Ly6G	BV421	1A8	Biolegend
Lyve1	AF660	ALY7	eBiosciences
MHCII IIA/IE	APCe780	M5/114.15.2	eBiosciences
RELMa	Unconjugated	Rabbit polyclonal	PeproTech
Siglec F	BV421	E50-2440	BD
TCRb	BV421	h57-597	Biolgend
Tim4	PE	RMT4-54	Biolegend
	APC		
**Anti-Human antibodies**			
CD3	PE	HIT3a	Biolegend
CD11b	AF700	M1/70	Biolegend
CD14	e450	HCD14	Biolegend
CD16	BV711	3G8	Biolegend
CD19	PE	HIB19	Biolegend
CD45	BV650	HI30	Biolegend
CD64	APCcy7	10.1	Biolegend
CD62L	PerCPcy5	DREG-56	Biolegend
CD163	BV605		Biolegend
CD206	AF647	M1/70	Biolegend
HLA-DR	FITC	L243	Biolegend
NCAM	PE	MEM188	Biolegend
**Secondary antibody**			
Goat-anti Rabbit Ig	Xenon-488		Invitrogen
Goat-anti Rat Ig	APC		Biolegend

Table S1. List of antibodies used in the study.
