# Supplementary material for: Role of Tim4 in the regulation of ABCA1+ adipose tissue macrophages and post-prandial cholesterol levels
Source: Nat Commun. 2021 Jul 21;12:4434. doi: 10.1038/s41467-021-24684-7 (PMC8295389; doi:10.1038/s41467-021-24684-7)
Supplement: Supplementary file 3 — Description of Additional Supplementary Files [file 41467_2021_24684_MOESM3_ESM.docx]

Description of Additional Supplementary Files

Title: Supplementary Data 1

Description: List of conserved DEGs for each cluster

Title: Supplementary Data 2

Description: List of DEGs between cluster 1 and cluster 4.

Title: Supplementary Data 3

Description: List of DEGs between HFD and CD conditions for each cluster.

Title: Supplementary Data 4

Description: List of GOBP terms identified for DEGs between HFD and CD conditions

Title: Supplementary Data 5

Description: List of DEGs between cluster 8 and cluster 4.
