# Supplementary material for: Role of Tim4 in the regulation of ABCA1+ adipose tissue macrophages and post-prandial cholesterol levels
Source: Nat Commun. 2021 Jul 21;12:4434. doi: 10.1038/s41467-021-24684-7 (PMC8295389; doi:10.1038/s41467-021-24684-7)
Supplement: Supplementary file 9 — Reporting Summary [file 41467_2021_24684_MOESM9_ESM.pdf]

nature research

Corresponding author(s): Cecile Benezech

Last updated by author(s): 10.06.2021

Reporting Summary

Nature Research wishes to improve the reproducibility of the work that we publish. This form provides structure for consistency and transparency in reporting. For further information on Nature Research policies, see our Editorial Policies and the Editorial Policy Checklist.

Statistics

For all statistical analyses, confirm that the following items are present in the figure legend, table legend, main text, or Methods section.

n/a	Confirmed	
☐	☒	The exact sample size (*n*) for each experimental group/condition, given as a discrete number and unit of measurement
☐	☒	A statement on whether measurements were taken from distinct samples or whether the same sample was measured repeatedly
☐	☒	The statistical test(s) used AND whether they are one- or two-sided<br>*Only common tests should be described solely by name; describe more complex techniques in the Methods section.*
☐	☒	A description of all covariates tested
☐	☒	A description of any assumptions or corrections, such as tests of normality and adjustment for multiple comparisons
☐	☒	A full description of the statistical parameters including central tendency (e.g. means) or other basic estimates (e.g. regression coefficient) AND variation (e.g. standard deviation) or associated estimates of uncertainty (e.g. confidence intervals)
☒	☐	For null hypothesis testing, the test statistic (e.g. *F*, *t*, *r*) with confidence intervals, effect sizes, degrees of freedom and *P* value noted<br>*Give P values as exact values whenever suitable.*
☒	☐	For Bayesian analysis, information on the choice of priors and Markov chain Monte Carlo settings
☒	☐	For hierarchical and complex designs, identification of the appropriate level for tests and full reporting of outcomes
☒	☐	Estimates of effect sizes (e.g. Cohen's *d*, Pearson's *r*), indicating how they were calculated

Our web collection on statistics for biologists contains articles on many of the points above.

Software and code

Policy information about availability of computer code

Data collection	Flow-data samples were acquired using FACSDiva software 6.3.1, BD Biosciences.<br>Confocal microscopy data were acquired using Leica LAS X software v3.5.7.23225.<br>Sequencing datasets were acquired using NovaSeq S1 Illumina.
Data analysis	All Flow data were analyzed using FlowJo 10 software (Tree Star).<br>Confocal images were analyzed using LAS-X-3D (Leica) v3.5.7.23225 and IMARIS software (2018).<br>Sequence reads were processed using Cell Ranger v3.0.2 single-cell software Suite from 10x Genomics.<br>Sc-RNAseq was analyzed using Seurat R package version 3.1.1.<br>R software version 3.1.1 was used for all bioinformatics analyses.<br>g:Profiler was used for pathway analysis.<br>Microsoft excel version 16.15 was used to organised tabulated data.<br>All statistical analyses were performed using GraphPad Prism 7.0b.

For manuscripts utilizing custom algorithms or software that are central to the research but not yet described in published literature, software must be made available to editors and reviewers. We strongly encourage code deposition in a community repository (e.g. GitHub). See the Nature Research guidelines for submitting code & software for further information.

Data

Policy information about availability of data

All manuscripts must include a data availability statement. This statement should provide the following information, where applicable:

- Accession codes, unique identifiers, or web links for publicly available datasets
- A list of figures that have associated raw data
- A description of any restrictions on data availability

The authors declare that all relevant data supporting the finding of this study are available on request. R scripts for performing the main steps of analysis are available from the corresponding authors on reasonable request. scRNA-seq data sets have been deposited at GEO: GSE168278.

Field-specific reporting

Please select the one below that is the best fit for your research. If you are not sure, read the appropriate sections before making your selection.

- [x] Life sciences
- [ ] Behavioural & social sciences
- [ ] Ecological, evolutionary & environmental sciences

For a reference copy of the document with all sections, see nature.com/documents/nr-reporting-summary-flat.pdf

Life sciences study design

All studies must disclose on these points even when the disclosure is negative.

Sample size

Sample sizes were not calculated prior to performing experiments but conform to standard practices; For animal experiments, n=5 and number of repeats were chosen based on the previous publications in the field (Hill et al., 2018; Jaitin et al., 2019). Sample sizes were sufficient to detect differences between groups.

Data exclusions

No data were excluded from analysis. Some samples were lost during processing and thus could not be included.

Replication

Figure 1: Data pooled from n=4,358 cells from n=6 mice (3 mice kept on control diet and 3 mice fed overnight with high fat diet).
Figure 2B: Representative data from 8 mice analyzed in two independent experiments.
Figure 2C-D: Data pooled from three independent experiments with n=15 mice per group.
Figure 2E-J: Data pooled from two independent experiments with n=9-12 mice per group.
Figure 3A and 3E: Same dataset as Figure 1.
Figure 3C-D: Data pooled from two independent experiments with n=7 mice.
Figure 3F: Representative data from 8 mice analyzed in two independent experiments.
Figure 4A: Representative data from 8 mice analyzed in two independent experiments.
Figure 4B: Representative data from 4 patients analysed separately.
Figure 4C: Representative data from 4 mice analyzed in two independent experiments.
Figure 5: Same dataset as Figure 1.
Figure 6B: Data representative of three independent experiments with n=5 mice per group.
Figure 6C, D, E, F, G, H, and J: Data pooled from two to three independent experiments n=8 to 15 mice per group.
Figure 6I: Data pooled from n=3 biological replicates per group.
Figure 7A-B: Data pooled from two independent experiments with n=6 to 8 mice per group.
Figure 7D: Data pooled from two independent experiments with n=8 mice per group.
Figure 7E-H: Data pooled from two independent experiments with n=8 mice per group.
Figure S1: Same dataset as Figure 1.
Figure S2A-B: Same dataset as Figure 2C.
Figure S2C-D: Same dataset as Figure 2E.
Figure S2E: Data pooled from two independent experiments with n=8 mice per group.
Figure S3: Representative data from 3 patients analysed separately.
Figure S5: Data pooled from one experiment with n=4 mice per group. Data representative of 2 independent experiments.
Figure S5C: Data representative of two independent experiments.
Figure S5D: Same dataset as Figure 7B.

Randomization

For animal experiments, mice were randomly allocated to treatment groups. Whenever possible, treatments were spread across animal cages.

Blinding

Due to the nature of the treatment, which is recognizable (diet), investigators were not blinded to group allocations. Whenever possible, investigators were blinded for data acquisition and analysis.

Reporting for specific materials, systems and methods

We require information from authors about some types of materials, experimental systems and methods used in many studies. Here, indicate whether each material, system or method listed is relevant to your study. If you are not sure if a list item applies to your research, read the appropriate section before selecting a response.

Materials & experimental systems

n/a	Involved in the study	
	x	Antibodies
x		Eukaryotic cell lines
x		Palaeontology and archaeology
	x	Animals and other organisms
	x	Human research participants
x		Clinical data
x		Dual use research of concern

Methods

n/a	Involved in the study	
x		ChIP-seq
	x	Flow cytometry
x		MRI-based neuroimaging

Antibodies

Antibodies used

Antigen Name / Conjugate / Clone / cat number / Manufacturer / titration

Anti-mouse antibodies
ABCA1 Alexa-647 5A1-1422 MCA2681A647 Bio-Rad
CD11b PE Dazzle M170 101256 Biolegend (0.2mg/ml) 1:200
CD11b VioBlue REA592 130-113-803 Miltenyi (1:200)
CD11c BV605 N418 117334 Biolegend (0.2mg/ml) 1:200
CD11c APC-Vio770 REA754 130-110-837 Miltenyi
CD115 (CSF1R) APC 135509 Biolegend (0.2mg/ml)
CD19 BV421 6D5 115537 Biolegend (0.2mg/ml) 1:200
CD36 PE REA1184 130-122-084 Miltenyi
CD206 FITC MCA2235FA (discontinued) Miltenyi (0.2mg/ml) 1:200
CD45.1 PE/Cy7 A20 110729 Biolegend (0.2mg/ml) 1:200
CD45.2 BV450 104 48-0454-82 eBiosciences (0.2mg/ml) 1:200
CD45.2 BV650 104 109835 Biolegend (0.2mg/ml) 1:200
F4/80 PE/Cy7 BM8 123114 Biolegend (0.2mg/ml) 1:200
F4/80 FITC REA126 130-116-499 Miltenyi 1:20
HSP-70 (HSP1A1) FITC 130-126-011 REA349Miltenyi
Ki-67 FITC REA183 130-117-691 Miltenyi 1:200
LAMP1 PE REA792 130-102-219 Miltenyi 1:200
LAMP2 PE M3/84 (discontinued) Miltenyi 1:200
Ly6C AF700 HK1.4 128024 Biolegend (0.5 mg/ml) 1:200
Ly6G BV421 1A8 127627 Biolegend (0.2mg/ml) 1:200
Lyve1 AF660 ALY7 50-0443-82 eBiosciences 1:200
MHCII IIA/IE APCe780 M5/114.15.2 47-5321-82 eBiosciences (0.2mg/ml) 1:600
RELMa Unconjugated Rabbit polyclonal 500-P214 PeproTech 1:300
Siglec F BV421 E50-2440 562681 BD (0.2mg/ml) 1:200
TCRb BV421 h57-597 109230 Biolegend (0.2mg/ml) 1:200
Tim4 PE RMT4-54 130006 Biolegend (0.2mg/ml) 1:1200
Tim4 APC RMT4-54 130008 Biolegend (1:50)

Anti-Human antibodies
CD3 PE HIT3a 300308 Biolegend (1:40)
CD11b AF700 M1/70 101222 Biolegend (1:200)
CD14 e450 HCD14 325602 Biolegend (1:25)
CD16 BV711 3G8 302002 Biolegend (1:20)
CD19 PE HIB19 302208 Biolegend (1:40)
CD45 BV650 HI30 304043 Biolegend (1:20)
CD64 APCcy7 10.1 305026 Biolegend (1:20)
CD62L PerCPcy5 DREG-56 304824 Biolegend (!:20)
CD163 BV605 333616 Biolegend (1:20)
CD206 AF647 M1/70 101218 Biolegend (1:20)
HLA-DR FITC L243 307604 Biolegend (1:20)
NCAM PEMEM188 Biolegend (1:40)
Secondary antibody
Goat-anti Rabbit Ig Xenon-488 A48282 Invitrogen (1:400)
Goat-anti Rat Ig APC 405407 Biolegend (1:400)

Validation

All antibody used have been tested and validated for their target species (mouse or human) and the application (Flow-cytometry and/or immuno-fluorescence) as stated on the website of the manufacturers.
https://www.bio-rad-antibodies.com/our-antibody-validation-principles.html

https://www.biolegend.com
https://www.miltenyibiotec.com/GB-en/products/macs-antibodies/antibody-validation.html

Antibodies were used at a concentration per recommendation by the respective manufacturer's website, or following in-house titration, to identify highest stain index. All antibodies were validated by us using appropriate FMO/isotype controls.

Animals and other organisms

Policy information about studies involving animals; ARRIVE guidelines recommended for reporting animal research

Laboratory animals	Experiments were performed using male C57BL/6 (C57BL/6JOlaHsd) mice aged 8-12 weeks. All animals were bred and housed at 22–23 °C on a 12 h light/dark cycle with free access to water and food under specific pathogen–free conditions at the University of Edinburgh Animal Facilities.
Wild animals	The study did not involved wild animals.
Field-collected samples	The study did not involved samples collected from the fields.
Ethics oversight	All experiments were conducted under a license granted by the Home Office (UK) that was approved by the University of Edinburgh animal welfare and ethics review board. All individual experimental protocols were approved by a named veterinarian surgeon prior to the start of the experiment.

Note that full information on the approval of the study protocol must also be provided in the manuscript.

Human research participants

Policy information about studies involving human research participants

Population characteristics	Patients undergoing laparoscopic surgery under general anaesthesia for hernia repair or gastric bypass (2 females, 1 male with age between 49 and 56).
Recruitment	Potential participants were recruited from scheduled elective abdominal operating theatre lists at the Royal Infirmary of Edinburgh , Edinburgh UK.
Ethics oversight	The human study was done in compliance with all relevant ethical regulations; following approval by the East of Scotland Research Ethics Service REC 1 (15/ES/0094); with all patients providing written informed consent prior to any study procedures.

Note that full information on the approval of the study protocol must also be provided in the manuscript.

Flow Cytometry

Plots

Confirm that:

- [x] The axis labels state the marker and fluorochrome used (e.g. CD4-FITC).
- [x] The axis scales are clearly visible. Include numbers along axes only for bottom left plot of group (a 'group' is an analysis of identical markers).
- [x] All plots are contour plots with outliers or pseudocolor plots.
- [x] A numerical value for number of cells or percentage (with statistics) is provided.

Methodology

Sample preparation	Murine gonadal adipose tissue were enzymatically digested with 1mg/ml Collagenase D (Roche) for 35 minutes at 37oC in RPMI 1640 (Sigma) containing 1% Fetal Bovine Serum (FBS) (Sigma). Peritoneal exudate cells (PEC) were isolated by flushing murine peritoneal cavities with RPMI 1640 (Sigma). The liver was perfused before dissection with 5 ml of RPMI 1640 (Sigma) injected through the portal vein. The tissue was cut into small pieces and homogenized using the gentleMACS dissociator (Miltenyi) in buffer containing Collagenase 2 (Sigma 0.425 mg/ml), Collagenase D (Roche 0.625 mg/ml) Dispase (Gibco 1mg/ml) and DNase (Roche 30µg/ml). After for 20 minutes incubation at 37C, the tissue was homogenized further using the dissociator. Red blood cells were lysed using red blood cell lysis buffer (Sigma).<br>Human AT was weighed and ± 0.500 g of tissue was digested using 2mg/ml Collagenase I (Worthington) in PBS (Invitrogen/sigma) 2% Bovine Serum Albumin (BSA, Sigma), samples were disrupted using an Octolyser (Miltenyi), incubated at 37oC with intermittent shaking for 45 minutes, subjected to a second Octolyser dissociation step, ions were chelated by addition of EDTA (0.5M, Sigma), samples were filtered through a 100µM filter (BD) and washed with 20ml of 2%BSA PBS prior to centrifugation at 1700pm for 10 minutes. The cell pellet was resuspended in 2ml of PBS 2% BSA for flow-cytometric analysis.
Instrument	Flow-data was acquired using a BD Fortessa LS6.<br>Flow sorting was performed using a BD FACsAria 2
Software	All Flow data were analysed using FlowJo 10 software (Tree Star).

Cell population abundance

Abundances of the cell-sorted population for scRNAseq analysis is stated in the manuscript. Cells were sorted as single cells, DAPI-CD45+TCRb-CD19-SiglecF-Ly6G-CD11b+F4/80+. The purity was >95%. The identity of the cells was than determined based on single-cell transcriptomics.

Gating strategy

Figure 1: EAT were analysed by dead cell exclusion, determination of single cell populations using scatter profiles and based on CD45 positivity. CD19+ B-cells, TCRβ+ T cells, Siglec F+ Eosinophils and Ly6G+ neutrophils were excluded. The remaining CD11b+ were gated followed by determination of Ly6C-F4/80+ macrophages for cell-sorting.

Figure 2A-B: EAT were analysed by dead cell exclusion, determination of single cell populations using scatter profiles and based on CD45 positivity. CD19+ B-cells, TCRβ+ T cells, Siglec F+ Eosinophils and Ly6G+ neutrophils were excluded. The remaining Ly6C-F480+ were gated followed by gating of F4/80highCd11bint and F4/80low macrophages and determination of F4/80highTim4+ and F4/80highTim4- macrophage populations in the F4/80highCD11bint gate. The profiles of staining for Lyve1, MHCII, CD206, RELMa and CSF1R were analysed.

Figure 2C: EAT were analysed by dead cell exclusion, determination of single cell populations using scatter profiles and based on CD45 positivity. CD19+ B-cells, TCRβ+ T cells and Ly6G+ neutrophils were excluded. Eosinophils were gated as CD11b +SiglecF+, monocytes as CD11b+Ly6C+ and all macrophages as CD11b+Ly6C-F480+. Chimerism was determined as shown in Figure S2A by gating on CD45.2intCD45.1- cells.

Figure 2D: F4/80highTim4+, F4/80highTim4- and F4/80low macrophage populations were gated as described in Figure 2A and chimerism was determined as shown in Figure S2B by gating on CD45.2intCD45.1- cells.

Figure 2F: EAT were analysed by dead cell exclusion, determination of single cell populations using scatter profiles and based on CD45 positivity. CD19+ B-cells, TCRβ+ T cells, Siglec F+ Eosinophils and Ly6G+ neutrophils were excluded. The remaining Ly6C-F480+ macrophages were gated followed by gating of F4/80highCD11bint and F4/80lowCD11bhigh macrophages and determination of F4/80highTim4+ and F4/80highTim4- populations in the F4/80highCD11bint gate and the gating of F4/80lowCD11c+ and F4/80lowCD11c- populations in the F4/80lowCD11bhigh gate as shown in Figure S2C. Chimerism was determined by gating CD45.2intCD45.1-.

Figure 2J: Ki67+ cells were gated on the macrophage populations gated as in 2F.

Figure 3C and 3F: The profiles of staining for LAMP2, Lysotracker, LipidTox, CD36 and ABCA1 were analysed on the macrophage populations gated as in Figure 2A.

Figure 6B: EAT were analysed by dead cell exclusion, determination of single cell populations using scatter profiles and based on CD45 positivity. F4/80highCd11bint and F4/80low macrophages were gated and F4/80highTim4+ and F4/80highTim4- macrophage populations were determined in the F4/80highCD11bint gate. Anti-Tim4 clone RMT4-54 directly conjugated was used to identify Tim4+ cells. This was the same clone used for in vivo Tim4 blockade.

Figure 6F, G, H and J: The profile of expression of Lysotracker, ABCA1, CD36 and HSP70 was analysed on the macrophage populations gated as in Figure 6B. Anti-Tim4 clone RMT4-54 was used to identify Tim4+ cells followed by anti-Rat-Alexa 647 to detect anti-Tim4. This enabled detection of Tim4+ cells even in animals which received anti-Tim4 blockade treatment.

Figure I6: F4/80highTim4+ macrophages were gated as in Figure 6F for cell-sorting,

Figure 7A-B: EAT, PEC and liver were analysed by dead cell exclusion, determination of single cell populations using scatter profiles and based on CD45 positivity. CD19+ B-cells, TCRβ+ T cells, Siglec F+ Eosinophils and Ly6G+ neutrophils were excluded. The remaining CD11b+ cells were gated followed by gating of F4/80+Tim4+ macrophages as shown in Figure 7C. The profiles of staining for LAMP1 and ABCA1 were analysed.

Figure S2E: Macrophages were gated as in Figure 2A.

Figure S3: AT were analysed by dead cell exclusion, determination of single cell populations using scatter profiles and based on CD45 positivity. CD19+ B-cells, CD3+ T cells, NCAM+ NK cells were excluded. The remaining CD11b+ cells were gated followed by gating of CD16+CD14-, CD16+CD14+ and CD16-CD14+ cell populations. The profiles of staining for Tim4, CD163, CD206, CD64, CD62L and HLA-DR were analysed.

Figure S5A-B: F4/80highTim4+ macrophages were gated as in 6F. The profiles of BODIPY-LDL and lysotracker staining were analysed.

Figure S5C: BODIPY-LDL was analysed using scatter profiles and based on BODIPY-LDL positivity. Chylomicrons was analysed using scatter profiles. The profile of staining for Annexin V was determined.

Figure S5D: PEC macrophages were gated as in Figure 6A. The profile of staining for ABCA1 was determined in Tim4+ and Tim4- macrophage populations.

☒ Tick this box to confirm that a figure exemplifying the gating strategy is provided in the Supplementary Information.
